# RECUR: identifying recurrent amino acid substitutions from multiple sequence alignments

**DOI:** 10.1093/molbev/msag036

**Published:** 2026-02-10

**Authors:** Elizabeth H J Robbins, Yi Liu, Steven Kelly

**Affiliations:** Department of Biology, University of Oxford, Oxford OX1 3RB, UK; Department of Biology, University of Oxford, Oxford OX1 3RB, UK; Department of Biology, University of Oxford, Oxford OX1 3RB, UK

**Keywords:** recurrent evolution, convergent evolution, parallel evolution, adaptation, phylogeny, multiple sequence alignment, SARS-CoV-2 surface glycoprotein

## Abstract

Identifying recurrent changes in biological sequences is important to multiple aspects of biological research—from understanding the molecular basis of convergent phenotypes, to pinpointing the causative sequence changes that give rise to antibiotic resistance and disease. Here, we present RECUR, a method for identifying recurrent amino acid substitutions from multiple sequence alignments that is fast, easy to use, and scalable to thousands of sequences. We demonstrate that RECUR's recurrence detection achieves 100% accuracy on simulated data with known evolutionary histories. We further show that RECUR is robust to realistic levels of tree inference error. Finally, we apply RECUR to a large set of surface glycoprotein (S) protein sequences from SARS-CoV-2. This analysis identified widespread recurrent evolution throughout the protein with significant enrichment in the exposed receptor-binding S1 subunit and at the interface with the human angiotensin-converting enzyme 2 (hACE2). In contrast, recurrent substitutions were depleted at the trimeric interface of the S protein. *In silico* modelling showed that recurrent substitutions had no directional effect on stability at either interface, but effects at the hACE2 interface were significantly more variable. Multiple substitutions with large destabilizing effects on hACE2 binding have been linked to immune escape, while others represented reversions back to the reference sequence, suggesting that recurrent evolution at this interface reflects opposing selective pressures balancing receptor binding with immune evasion. A standalone implementation of the algorithm is available under the GPLv3 license at https://github.com/OrthoFinder/RECUR.

## Introduction

Recurrent evolution arises when the same biological innovation evolves independently on multiple occasions. It is found across the tree of life and can be observed across multiple phenotypic scales, from whole organisms down to the molecular level (Stern 2013). At all phenotypic levels, recurrent evolution can provide insight into the role that natural selection plays in overcoming environmental constraints. Paradigm examples at larger phenotypic scales include the independent evolution of wings in pterosaurs, insects, bats, and birds (Hunter 2007); the independent evolution of high-resolution camera-like eyes in vertebrates, cephalopods, and arthropods (Nilsson 2013); and the independent evolution of the C_4_ carbon concentrating mechanism in plants (Sage 2004). At the molecular level, recurrent phenotypic evolution can be observed as repeated amino acid changes in proteins, reflecting selective pressures on protein function. Studying these recurrent molecular changes can help uncover the mechanistic basis of disease (Ingram 1957; Cutting et al. 1990; Davies et al. 2002; Olivier et al. 2010; Jänne et al. 2022), how proteins change to better suit environmental conditions (Bull et al. 1997; Christin et al. 2007; Christin et al. 2008; Yokoyama et al. 2008; Christin et al. 2009; Liu et al. 2010; Projecto-Garcia et al. 2013; van Ditmarsch et al. 2013; He et al. 2021; Robbins and Kelly 2024), and how organisms defend against toxic molecules (including antimicrobials, antivirals, herbicides, and pesticides) (Dobler et al. 2012; Feldman et al. 2012; Toprak et al. 2012; Zhen et al. 2012; Brodie III and Brodie Jr 2015; Ujvari et al. 2015; Iketani et al. 2023). Collectively, substantial insights into multiple biological phenomena at disparate scales can be gained by studying recurrent evolution.

**Figure 1:**
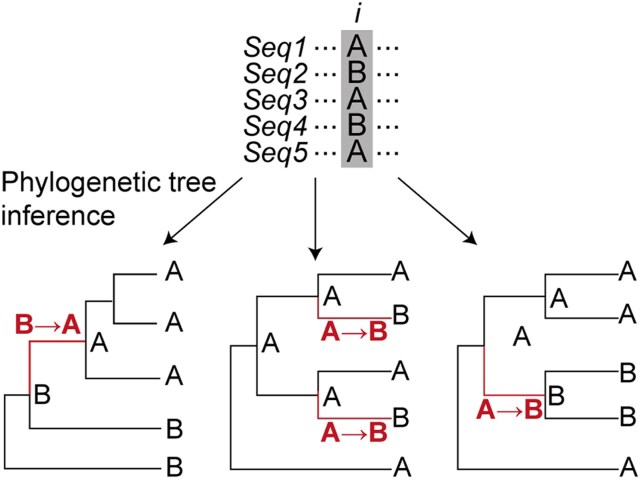
The evolutionary context of sequences in a multiple sequence alignment (MSA) dictates the substitutions inferred. A single site *i* from an MSA is shown on top with three alternative phylogenetic tree topologies below. Depending on the topology, different ancestral states are reconstructed at the internal nodes, leading to variation in the direction and frequency of substitutions.

In order to identify recurrent molecular evolution, biological sequences need to be analysed in the context of evolutionary history (Fig. 1). Phylogenetic analysis of sequence change allows the reconstruction of ancestral sequences (the internal nodes in the phylogenetic tree), enabling the direction and frequency of sequence change to be determined. Convergent and parallel evolution are both forms of recurrent evolution that can be inferred through such phylogenetic analyses. Convergent evolution refers to the independent acquisition of a trait from dissimilar ancestral traits, e.g. X → Y and Z → Y. Meanwhile, parallel evolution requires both the ancestral and descendent traits to be identical, e.g. multiple independent instances of X → Y. Here, the identity of the sequence change—whether arising from identical or non-identical ancestral states—is important and is independent of the rate at which the change occurs.

While some phylogenetic tools can identify individual substitutions when provided with a multiple sequence alignment and a precomputed phylogenetic tree, there remains a lack of dedicated methods for systematically detecting and quantifying recurrent molecular evolution directly from alignments. Here, we present RECUR, a phylogenetic tool designed to address this gap by identifying recurrent substitutions, specifically parallel substitutions, that have occurred in a protein or codon multiple sequence alignment. RECUR takes a multiple sequence alignment as input, infers the phylogenetic tree if one is not supplied, and identifies all recurrent sequence substitutions present within the evolutionary history of that alignment and their associated statistics. RECUR is fast, accurate and scalable to thousands of sequences, and we exemplify its utility on an alignment of 123,126 SARS-CoV-2 surface glycoprotein sequences.

## Results

### Workflow and overview of RECUR

RECUR was designed to identify recurrent amino acid substitutions that have arisen during the evolution of a set of protein sequences. RECUR requires as input a multiple sequence alignment of homologous protein sequences or a corresponding codon alignment. The method returns 1) the complete set of substitutions inferred to have occurred along the evolutionary history of the sequences in the alignment. 2) The complete set of substitutions that are recurrent, including whether any reversions (from the derived state back to the ancestral state) have also taken place. 3) A statistical analysis of the recurrent substitutions to identify those that have occurred more frequently than expected given the number of sequences, their phylogenetic relationship, and the underlying model of sequence evolution.

**Figure 2:**
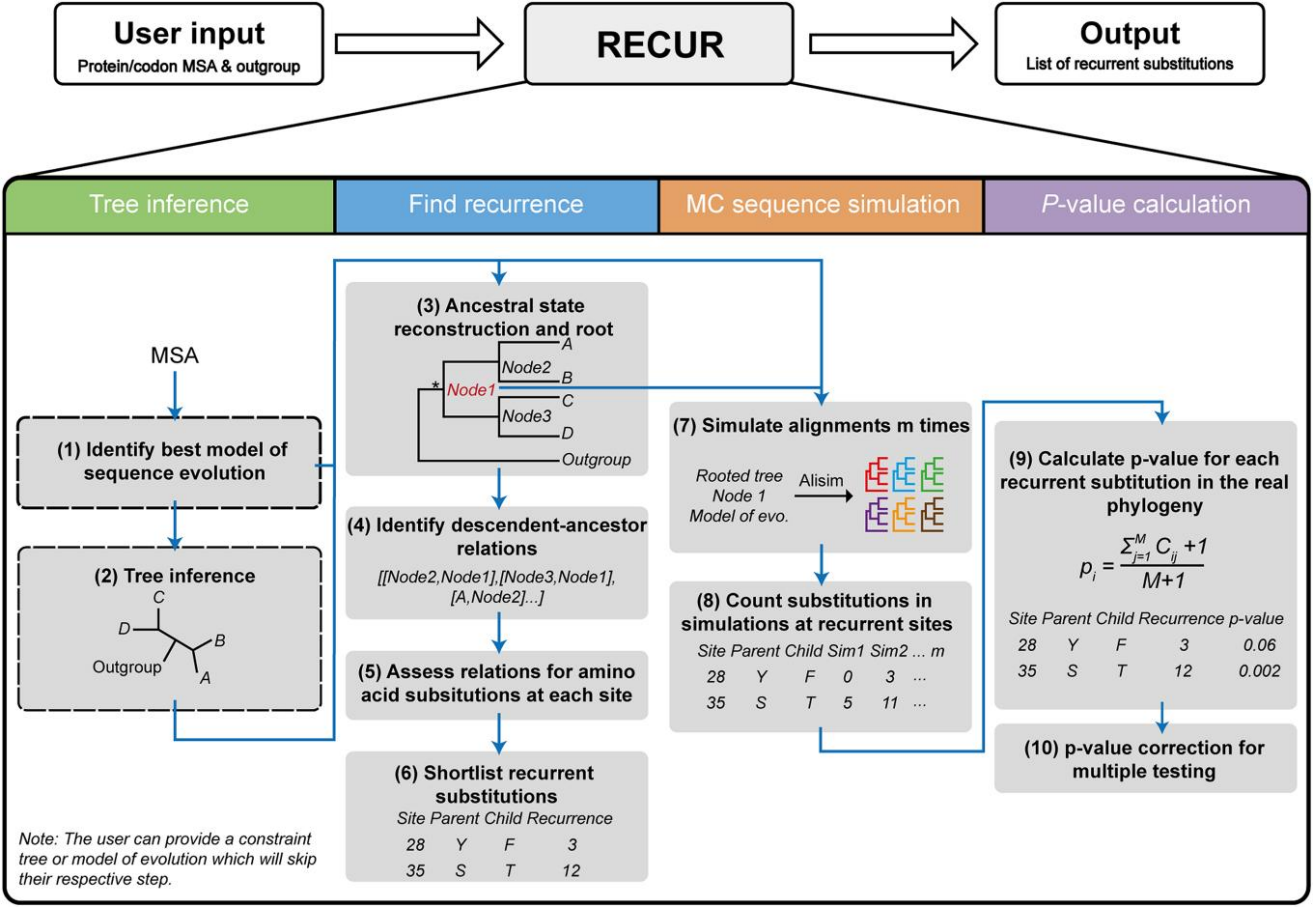
Overview of RECUR. The input (codon or protein) multiple sequence alignment is used to identify the best model of evolution (1), build a maximum likelihood phylogeny (2), and construct ancestral sequences (3). A user-defined outgroup is then used to root the tree (3) and assess each branch in the phylogeny for amino acid substitutions at each site in the protein sequence (4 and 5), from which recurrent substitutions are extracted (6). RECUR then performs a Monte Carlo (MC) simulation of sequence evolution, in which sequence evolution is then simulated *m* times using the topology of the inferred phylogeny, the best model of evolution and root sequence of the subtree of interest (*), which excludes the outgroup sequences (7). The recurrence of substitutions identified in step 6 is then assessed in each of the simulated alignments (8) and a *P*-value is calculated based on the number of simulations in which recurrence equals or exceeds that observed in the real alignment (9). Finally, *P*-values are adjusted for multiple testing (10).

An overview of the RECUR workflow is provided in Fig. 2. In brief, a phylogenetic tree is constructed from the input multiple sequence alignment and ancestral sequences are inferred for all internal nodes of the phylogenetic tree. All amino acid substitutions are then identified by assessing changes in the protein sequence along every branch in the phylogeny. Simulated multiple sequence alignments are then generated using the inferred ancestral sequence, the inferred tree, and the best-fitting model of sequence evolution and evaluated to identify those recurrent substitutions that have occurred more frequently than expected by chance (see Methods).

### RECUR accurately identifies recurrent substitutions in simulated multiple sequence alignments

To assess the accuracy of RECUR's recurrent detection algorithm, we simulated the evolution of 1,000 human protein-coding genes on randomly generated phylogenetic trees using phastSim (De Maio et al. 2022). We chose phastSim because it records the exact substitutions introduced during simulation, providing a known ground truth. Each simulated alignment, corresponding phylogenetic tree and ancestral sequences were provided directly to RECUR, such that this analysis specifically evaluated the correctness of RECUR's recurrence counting algorithm, rather than the performance of phylogenetic inference, model selection, or ancestral sequence reconstruction. The results were compared to the known history of events from the simulation to allow direct evaluation of its accuracy.

**Figure 3:**
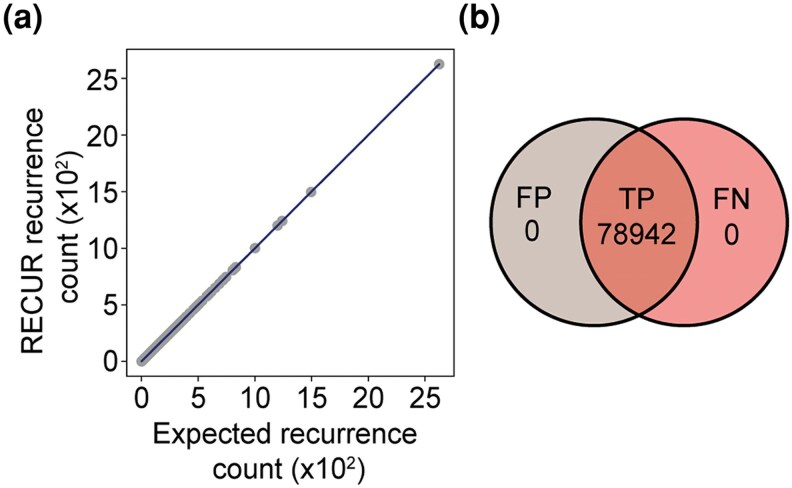
Assessing the accuracy of RECUR's recurrence detection algorithm. (a) Scatter plot showing the number of recurrent amino acid substitutions identified by RECUR versus the number introduced during sequence evolution by phastSim (expected recurrence count) across 1,000 simulations. (b) Venn diagram illustrating the overlap between recurrent substitutions introduced by phastSim and those detected by RECUR. True positives (TP) represent substitutions correctly identified with the exact recurrence frequency (e.g. A10V observed 5 times), false positives (FP) are substitutions detected by RECUR but not introduced by phastSim, and false negatives (FN) are substitutions introduced by phastSim but not detected by RECUR.

Across all simulated alignments, the RECUR algorithm correctly identified all recurrent substitutions (Fig. 3a and File S1). Moreover, RECUR correctly identified the exact recurrence counts (e.g. A10V occurred 5 times), with no false positives or false negatives (Fig. 3b). Together, these results establish that the RECUR's substitution counting algorithm can accurately and robustly detect recurrent substitutions from simulated sequence data.

### There is high concordance between RECUR and TreeTime in detecting recurrent substitutions

**Figure 4:**
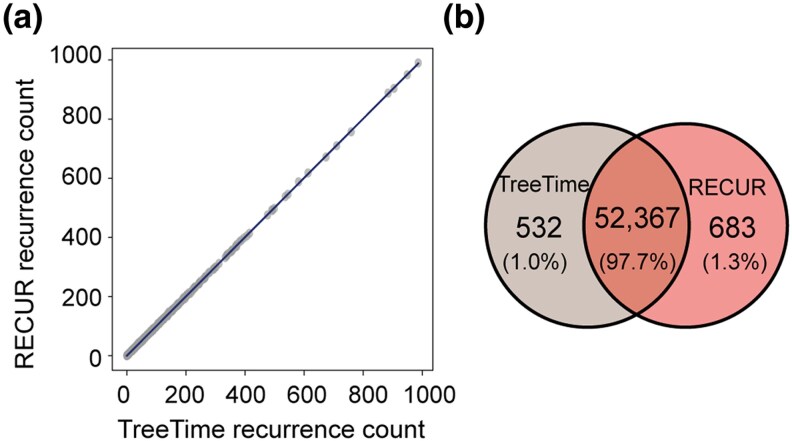
Comparing recurrence detection between RECUR and TreeTime. (a) Scatter plot showing the number of recurrent substitutions identified by TreeTime versus RECUR across 1,000 simulated alignments of human protein sequences. A linear regression line is provided. (b) Venn diagram illustrating the overlap between recurrent substitutions identified by RECUR and TreeTime with the correct frequency.

To further evaluate RECUR's performance, we benchmarked the method against a comparator method TreeTime (Sagulenko et al. 2018), which can infer substitutions across a phylogeny. Unlike RECUR, TreeTime does not directly report recurrence, but instead provides a list of substitutions inferred on each branch, from which the recurrence can be subsequently computed by a user. Both tools were applied to 1,000 randomly simulated human protein sequence datasets, enabling a direct comparison of the recurrent substitutions each tool identified. Across all simulated datasets, RECUR and post hoc analysis of TreeTime results showed a high degree of concordance in the number of recurrent substitutions detected (Fig. 4a). To quantify this agreement, we calculated the percentage overlap of all recurrent substitutions with the same frequency identified by both methods. RECUR and TreeTime exhibited a mean overlap of 97.7% (Fig. 4b and File S2). Notably, all observed differences were attributable to differences in the ancestral state reconstruction. RECUR leverages IQ-TREE for ancestral state reconstruction, a widely used and well-validated phylogenetic software, whereas TreeTime implements its own ancestral state reconstruction method. When ancestral states were identical, RECUR and TreeTime produced identical results (File S3). Thus, RECUR achieves high concordance with TreeTime while providing a streamlined, automated approach for recurrence detection.

### The accuracy of phylogenetic tree construction influences recurrence detection

We next sought to investigate the effect of the accuracy of phylogenetic tree reconstruction on the detection of recurrent substitutions. To do this, we leveraged the inheritance properties of the angiosperm chloroplast genome, which contains 69 ubiquitously conserved single-copy protein-coding genes. The entire genome is inherited uniparentally as a single unit in the absence of recombination, such that all 69 ubiquitously conserved single-copy genes share an identical evolutionary history (Birky 1995; Mogensen 1996). While the phylogenetic tree inferred from a concatenated alignment of all 69 genes is the most likely evolutionary history of all genes, no single phylogenetic tree inferred from an individual gene's multiple sequence alignment is identical to this topology (Figure S1). Moreover, no two phylogenetic trees inferred from individual gene multiple sequence alignments are identical (Figure S2). Thus, this dataset provides a real-world example to assess the impact of real tree inference error on recurrence detection.

**Figure 5:**
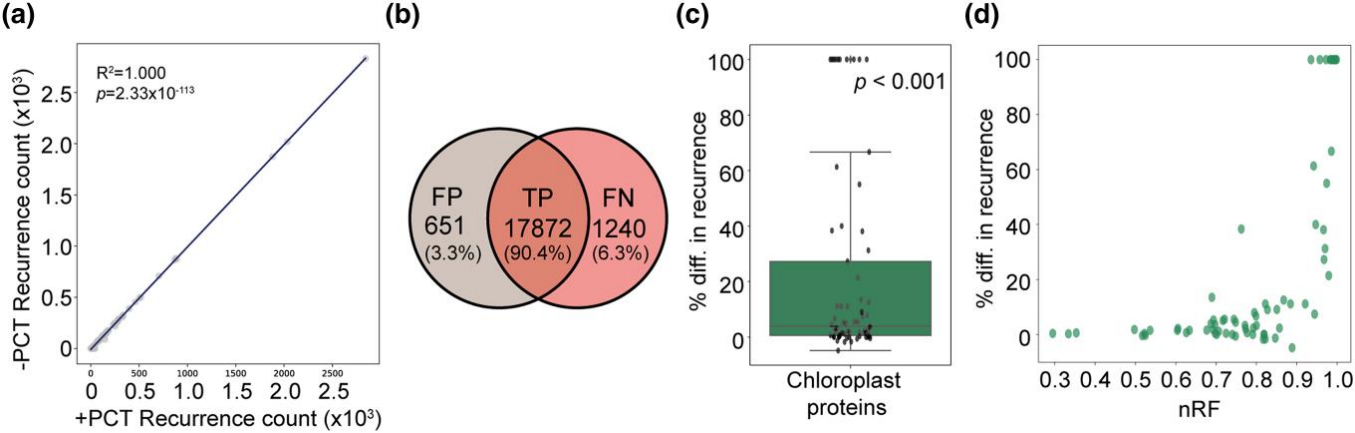
Assessing the effects of tree topology on recurrence detection by RECUR. (a) The total number of recurrent substitutions identified by RECUR for each of the 69 chloroplast-encoded proteins when using a gene tree inferred directly from the alignment (-PCT) versus a precomputed constraint species tree (+PCT). A linear regression line and associated statistics are provided. (b) Venn diagram showing the overlap between recurrent substitutions inferred using the species tree (+PCT) versus individual gene trees (−PCT). True positives (TP) are substitutions correctly identified with a gene tree, false positives (FP) are substitutions detected only with the gene tree, and false negatives (FN) are substitutions detected with the species tree but missed when using the gene tree. (c) Boxplot showing the percentage difference in recurrence count when the precomputed constraint species tree was provided versus when no constraint was used. The *P*-value shown represents the significance of a one-sample Wilcoxon test. (d) Scatter plot showing the relationship between the normalized Robinson-Foulds distance (nRF) and the percentage difference in recurrence count when the precomputed constraint species tree was provided versus when no constraint was used.

To assess the impact of tree inference error on recurrence detection, we compared RECUR’s results obtained using the phylogenetic tree inferred from the concatenated multiple sequence alignment with the results obtained using the phylogenetic tree inferred from each individual gene. This comparison revealed that the number of recurrent substitutions identified in each alignment was largely unaffected by realistic tree inference error (Fig. 5a). Across all alignments, 90.4% of detected recurrent substitutions were identical (Fig. 5b). Only 6.3% of recurrent substitutions were missed when using individual gene trees, and these tended to involve substitutions with lower recurrence levels (Fig. 5b). Similarly, only 3.3% of recurrent substitutions were false positives (Fig. 5b). Consistent with these results, introduction of tree inference error also reduced the counts for recurrent substitutions (Fig. 5c; Wilcoxon test, *P* < 0.001). Finally, there was a positive correlation between the topological distance between the gene tree and the species tree and the percentage difference in recurrence counts (Fig. 4d), showing that genes with more tree inference error exhibited greater differences in recurrence counts. Together, these results demonstrate that RECUR is robust to realistic levels of tree inference error with a high true positive rate, and low false positive and false negative rates. However, as for all analyses of evolution irrespective of the question under consideration, tree inference error can influence the results that are obtained.

### RECUR is fast and readily scalable to thousands of sequences

To demonstrate the runtime characteristics of RECUR, we evaluated the runtime and memory usage of the method across multiple sequence alignments ranging from 8 to 1,024 sequences. Each alignment comprised a randomly selected set of nonredundant SARS-CoV-2 surface glycoprotein (S protein) sequences (see Methods). Furthermore, to demonstrate the runtime characteristics of different steps of the method each alignment was subject to analysis with RECUR using eight threads under six distinct input options: 1) with no additional input and no bootstrap support for tree inference, 2) with no additional input but with bootstrap support calculations for the inferred tree, 3) with a precomputed best-fitting model of sequence evolution and no bootstrap support, 4) with a precomputed model and bootstrap support, 5) with a constraint tree, and 6) with both a constraint tree and a precomputed model of sequence evolution. For configurations 5 and 6, bootstrap support calculations were not performed because a constraint tree is provided.

**Figure 6:**
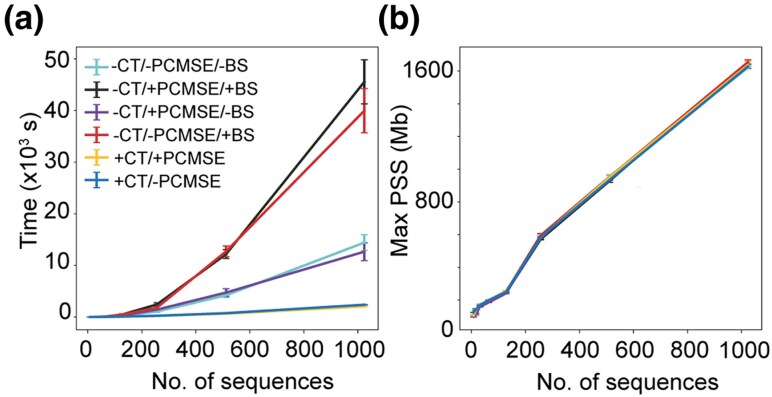
Analysing runtime and memory usage characteristics of RECUR. (a) Line graph showing the relationships between runtime and number of sequences in the protein multiple sequence alignment. The different lines indicate the six different input variations; cyan, no precomputed constraint tree, no precomputed model of sequence evolution and no branch support testing (-CT/-PCMSE/-BS); purple, no constraint tree, no branch support testing but with an evolutionary model (-CT/+PCMSE/-BS); red, no constraint tree, no evolutionary model but with branch support testing (-CT/-PCMSE/+BS); black, no constraint tree but both branch support testing and an evolutionary model (-CT/+PCMSE/-BS); blue, a constraint tree and no evolutionary model (branch support testing not applicable) (+CT/-PCMSE); and yellow a constraint tree and an evolutionary model (branch support testing not applicable) (+CT/+PCMSE). (b) Line graph showing the relationship between maximum proportional set size (PSS) with the number of sequences. Lines coloured as in (a).

The runtime is almost entirely consumed by tree inference (Fig. 6a) and thus RECUR was fastest when a constraint tree was provided, as this enables RECUR to skip this step. Runtime was also substantially reduced when bootstrap support calculations were omitted. In contrast, memory usage (Fig. 6b), measured as proportional set size (PSS), increased approximately linearly with the number of sequences and was unaffected by the inclusion of a constraint tree or evolutionary model. Thus, while runtime is significantly impacted by the requirement for tree inference and is further increased by the inclusion of branch support calculations, memory usage remains independent of the input conditions and scales linearly with alignment size.

### RECUR identifies widespread molecular recurrence during the evolution of the surface glycoprotein in SARS-CoV-2

**Figure 7:**
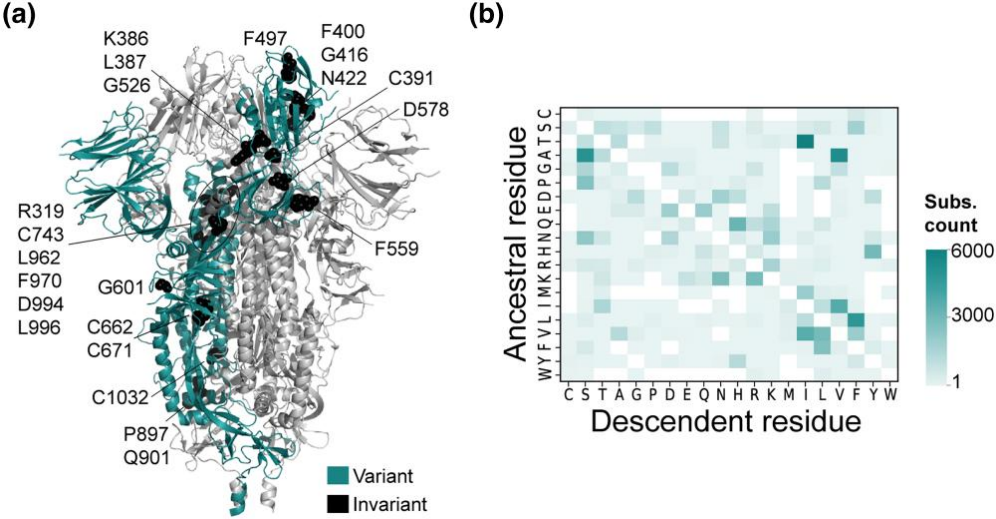
Position and type of amino acid substitutions inferred to have occurred by RECUR during the evolution of the S protein. (a) Structure of the closed S protein trimer (PDB: 6VXX) with variable residues (where amino acid substitutions have been inferred) coloured cyan and invariant residues (no inferred amino acid substitutions) shown as black spheres on a single chain. Labels and approximate positions for the invariant residues are provided. Two sites (633 and 974) with no inferred amino acid substitutions but with site deletions (not reported by RECUR), are omitted. The remaining two protomers of the trimer are coloured in light grey. (b) Heatmap of all amino acid substitutions identified by RECUR to have occurred in the S protein alignment across all sites. Rows signify the identity of the ancestral residue and columns signify the identity of the descendent residue.

**Figure 8:**
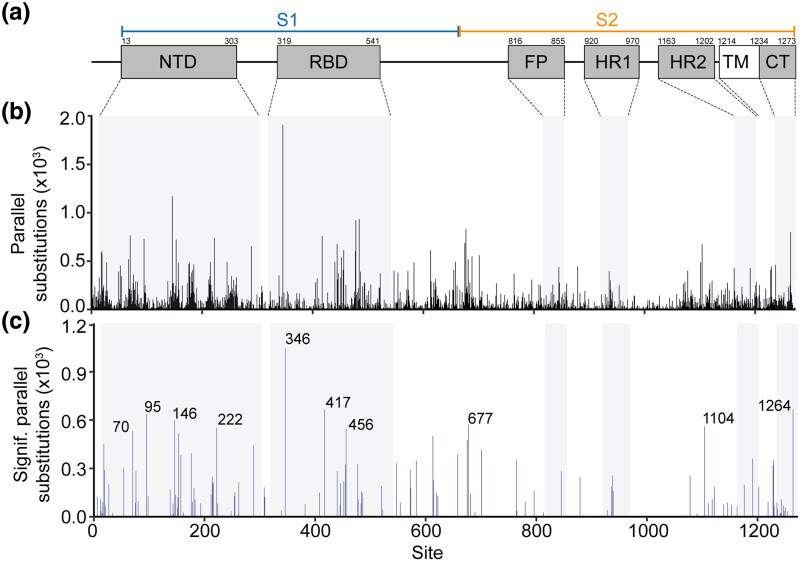
Recurrent evolution in the SARS-CoV-2 surface glycoprotein identified by RECUR. (a) Map showing positions of the S protein domains (UniProt accession: P0DTC2). Domains include N-terminal domain (NTD), receptor binding domain (RBD), fusion peptide (FP), heptapeptide repeat sequence 1 (HR1), heptapeptide repeat sequence 2 (HR2), transmembrane domain (TM), and cytoplasmic tail (CT). The position of the larger S1 (residues 14–685) and S2 (residues 686–1,273) subunits are indicated. (b) and (c) Bar charts showing the number of parallel substitutions and significant parallel substitutions (those not likely the result of stochastic mutation) at each site in S protein, respectively. Protein domains are highlighted. The top 10 sites with the most significant recurrent substitutions are labelled in (c).

To demonstrate the utility of RECUR on a real-world dataset, we applied the method to the complete set of nonredundant S protein sequences present in NCBI (*n* = 123,126, see Methods). The S protein, which forms a homotrimeric complex on the viral surface, is essential for host cell entry—mediating receptor recognition (predominantly the human angiotensin-converting enzyme 2, hACE2) via the exposed S1 subunit and facilitating membrane fusion through the more buried S2 subunit (Jackson et al. 2022). This sequence was chosen as an illustrative example due to: (1) the general interest in the evolution of this protein sequence as it is the target of many vaccines; (2) the surface location and biological role of the protein means that the sequence has been subject to selection for promoting transmissibility and immune system evasion (Carabelli et al. 2023) and thus is likely to have experienced substantial recurrent evolution (Korber et al. 2020; Wilkinson et al. 2022); and (3) the abundance of sequence data readily available in NCBI which can be subject to analysis. Application of RECUR to this dataset of S protein sequences identified 118,074 amino acid substitutions distributed across 98% (1,233/1,258) of aligned sites, with only 25 sites being invariant across all sequences in the alignment (Fig. 7a and File S4). A heatmap showing the frequency of the different types of amino acid substitutions inferred by RECUR is shown in Fig. 7b. Of the 118,074 substitutions, 97% (114,892/118,074) were recurrent and parallel and involved 5,879 distinct substitution patterns (File S5). The locations of these recurrent parallel substitutions were mapped onto the domains of the S protein (Fig. 8a, b), revealing a landscape of widespread recurrent evolution along the length of the protein.

To determine whether these recurrent substitutions have occurred more frequently than expected given the number of sequences, the model of evolution, and the underlying phylogenetic relationship between the sequences, RECUR ran a Monte Carlo simulation of sequence evolution. This revealed that 21% (24,325/114,892) of the recurrent substitutions identified above, which occurred at 132 different sites and included 172 distinct patterns of substitution, occurred significantly more frequently than expected (Fig. 8c and File S6). For illustrative purposes, the top 10 sites with the most recurrent substitutions deemed statistically significant are highlighted in Fig. 8c and Table 1. Eight of these sites are located in the exposed S1 region of the S protein, and include V70, T95, H146, and A222 in the N-terminal domain and R346, K417, and F456 in the receptor binding domain. All of these sites have all been associated with substitutions altering viral infectivity or immune escape (Table 1). For example, the R346T substitution (which occurred 243 times) increases S protein binding to the hACE2 receptor (Li et al. 2024). Interestingly, three of these sites, all of which are located in the S1 region, have experienced significant reversion substitutions (Table 1), indicating sequence space is being resampled—a phenomenon associated with cycles of selection pressures influencing viral protein evolution (Focosi et al. 2024b). This pattern of reversion suggests that regions of the S protein may be evolving within a constrained solution space, in which only a limited set of amino acid configurations are tolerated or advantageous under changing selective regimes. A wider examination of all significant recurrent substitutions revealed that 14% of significant recurrent substitutions (12 substitution pairs totalling 24 distinct substitution patterns) had a corresponding significant reversion substitution (File S6). In summary, RECUR detected widespread recurrent molecular evolution during the evolution of the S protein, including an abundance of reversion substitutions.

**Table 1:** The top 10 sites in the S protein with the most significantly recurrent parallel amino acid substitutions

Site	Region	Protein domain	Recurrent substitutions	Site annotation	Refs.
V70	S1	NTD	V→I V→F	Variations at site compensate for immune escape substitutions.	Meng et al. (2021)
T95	S1	NTD	T→I I→T	Mutations associated with immune escape and increased viral load.	Shen et al. (2021)
H146	S1	NTD	Q→K	In the NTD supersite for antibody binding. Mutations associated with immune escape.	McCallum et al. (2021b); Wang et al. (2023)
A222	S1	NTD	A→V	Mutations can alter ACE2 receptor binding (allosterically).	Ginex et al. (2022)
R346	S1	RBD	K→R R→T T→S T→R	Mutations associated with immune escape. Mutations can alter ACE2 binding affinity.	Li et al. (2024); Wang et al. (2024)
K417	S1	RBD	N→K K→N N→T	ACE2 receptor binding site. Mutations associated with immune escape.	Barton et al. (2021); Gupta et al. (2024)
F456	S1	RBD	F→L	Mutations associated with altered hACE2 binding.	Focosi et al. (2024a)
Q677	S1	…	Q→H	Mutations can alter membrane fusion capability. Mutations can alter furin protease activity given proximity to cleavage site 682-RRAR-685.	Zeng et al. (2021)
V1104	S2	…	V→L	Mutations may affect protein stability. Present in a T-cell epitope.	Li et al. (2024)
V1264	S2	CT	V→L	In 4.1, Ezrin, Radixin, and Moesin binding domain. Thus, it may affect subcellular trafficking of S protein.	Hu et al. (2023)

### There is a significant overlap between significantly recurrent sites and those evolving under positive selection

**Figure 9:**
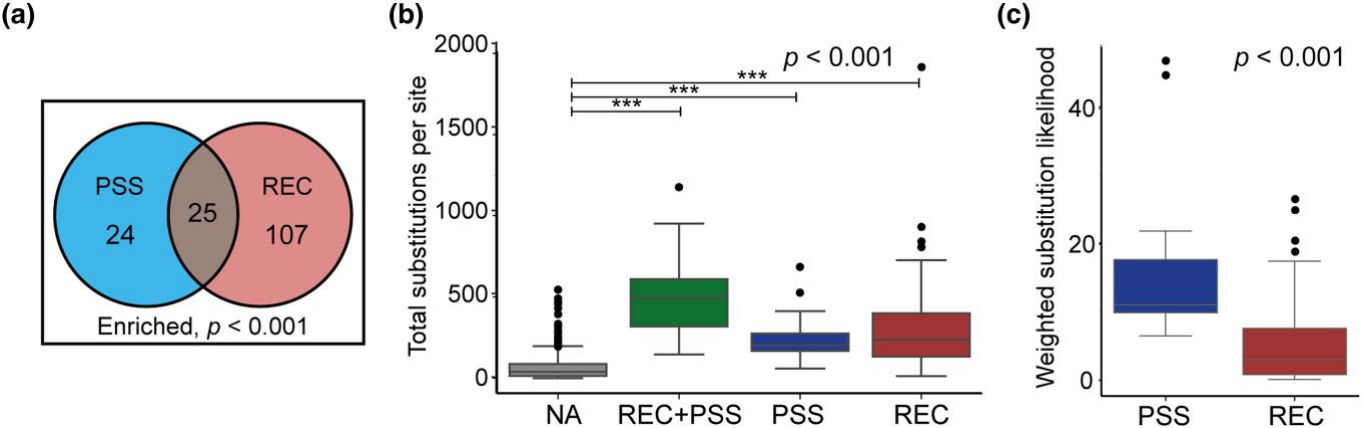
Comparison of sites under positive selection and sites with significant recurrent evolution identified by RECUR. (a) A Venn diagram showing the results of a hypergeometric test between sites under positive selection (PSS) (Ferreira et al. 2024) and those identified by RECUR as having significant recurrent evolution (REC). (b) Boxplot showing the total number of substitutions inferred to have occurred at sites under positive selection (PSS, *n* = 24), sites with significant recurrent evolution (REC, *n* = 107), both positive selection and significant recurrent evolution (REC + PSS, *n* = 25), and neither (nonadaptive, NA; *n* = 1,102). The *P*-value shown in the top right is the result of a Kruskal–Wallis test, and asterisks indicate the significance of a Dunn's post hoc test with Holm-Bonferroni *P*-value correction (*, *P* < 0.05; **, *P* < 0.01; ***, *P* < 0.001). (c) Boxplots showing the difference in the weighted average substitution likelihood occurring at sites exclusively under positive selection and sites exclusively under recurrent evolution. *P*-value shows the significance of a Wilcoxon rank-sum test.

Positive selection analyses have been extensively used to detect signatures of adaptive evolution by comparing synonymous and nonsynonymous substitution rates at individual sites. While this approach captures a distinct adaptive signature compared to that identified by RECUR, we sought to assess the degree of overlap between these two approaches. To do so, we compared sites identified as significantly recurrent by RECUR with those identified as evolving under positive selection based on multiple lines of evidence (Ferreira et al. 2024). We found that 51% of sites identified as evolving under positive selection were also identified by RECUR as exhibiting significant recurrent substitution, demonstrating a significant overlap between the two distinct analyses (hypergeometric test, *P* < 0.001; Fig. 9a and File S6). This comparison suggests that while the two approaches identify distinct signals of adaptive evolution, an enriched subset of sites exhibits both significant recurrence and elevated rates of nonsynonymous substitution.

Nevertheless, 49% of positive selection sites were not detected as recurrently evolving (Fig. 9a). Moreover, 81% of recurrently evolving sites were not detected as under positive selection (Fig. 9a). To better understand these differences, we compared the total number of substitutions occurring at sites detected by either method. This showed that nonadaptive sites—those detected by neither method—had the lowest numbers of substitutions, while sites identified by both or either method had the highest (Kruskal–Wallis test, *P* < 0.001; Fig. 9b). Thus, both methods detect elevated substitution rates.

Since RECUR assessed recurrence significance in the S protein using the likelihood of a substitution under the inferred model of protein evolution, we hypothesized that substitutions at sites exclusively identified under recurrent evolution would have lower likelihoods than those identified exclusively under positive selection, where significance is determined by the ratio of nonsynonymous and synonymous substitutions. To test this, we compared the weighted average likelihood of substitutions occurring at sites identified exclusively by either method (see Methods). This revealed that substitutions occurring at exclusively recurrently evolving sites had significantly lower likelihoods than those occurring at sites exclusively under positive selection (Wilcoxon test, *P* < 0.001; Fig. 9c). Overall, while there is strong concordance between RECUR and positive selection analyses in detecting adaptive evolution, key methodological differences result in additional unique contributions in identifying adaptive signatures by RECUR.

### Recurrent molecular evolution is enriched in the immune-exposed S1 subunit of the S protein

**Figure 10:**
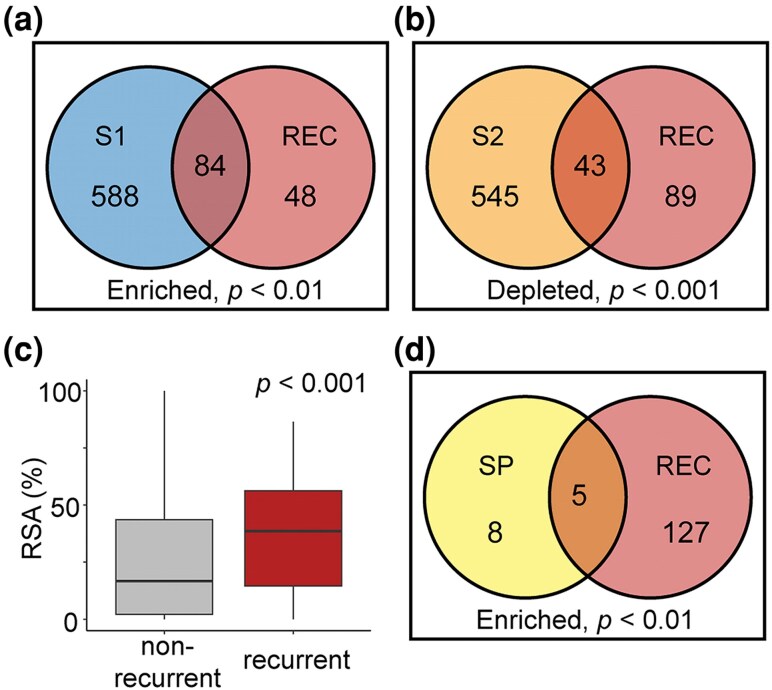
Assessing enrichment of recurrent evolution across regions of the S protein. (a and b) Venn diagrams showing the results of hypergeometric tests between the S1 and S2 subunits with significant recurrence sites (REC), respectively. (c) Boxplot showing the difference in the relative solvent accessibility (RSA) for sites identified as under significant recurrent evolution versus those that are not. The *P*-value indicates the significance of a Wilcoxon test. (d) A Venn diagram showing the results of the hypergeometric test between the signal peptide (SP) and significant recurrence sites.

We next sought to investigate the position within the S protein of the significant recurrent substitutions identified by RECUR. This revealed a significant enrichment in the number of sites that have undergone recurrent evolution within the S1 subunit, the region which mediates target recognition and receptor binding and is also the primary target of the immune system (hypergeometric test, *P* < 0.001; Fig. 10a). In contrast, there was a significant depletion in the number of recurrent sites located in the S2 subunit (hypergeometric test, *P* < 0.001; Fig. 10b), the region that facilitates membrane fusion and endosomal trafficking. In agreement with these results and with the increased exposure of the S1 subunit to the environment, sites that had undergone recurrent evolution exhibited significantly higher solvent accessibility compared to nonrecurrently evolving sites when mapped onto the closed structure of the trimeric S protein (Wilcoxon rank sum test, *P* < 0.001; Fig. 10c). These findings support that the S1 subunit, being more exposed on the viral surface, making it more accessible to the host immune system, has experienced stronger selective pressures compared to the S2 subunit.

For completeness, we also investigated the extent of recurrent evolution in the signal peptide, the N-terminal 13 amino acids upstream of the S1 region responsible for entry into the host's endocytic system. This revealed an enrichment of significant recurrent evolution within the signal peptide (hypergeometric test, *P* < 0.01; Fig. 10d). One of the recurrent substitutions identified in the signal peptide was S13I, which was inferred to have 37 occurrences across the phylogeny (File S6). The S13I substitution has been shown to shift the signal peptide cleavage site, leading to structural alterations to the N-terminal domain, interfering with the binding of some antibodies (McCallum et al. 2021a). Furthermore, S13I may increase the efficiency of protein secretion (Zhang et al. 2022). Thus, although often overlooked, the signal peptide of the S protein has hallmarks of having undergone adaptive evolution, with some substitutions already shown to influence viral replication and immune evasion.

### Recurrent molecular evolution has been restricted at the trimeric interface but enhanced at the hACE2 interface of the S protein

**Figure 11:**
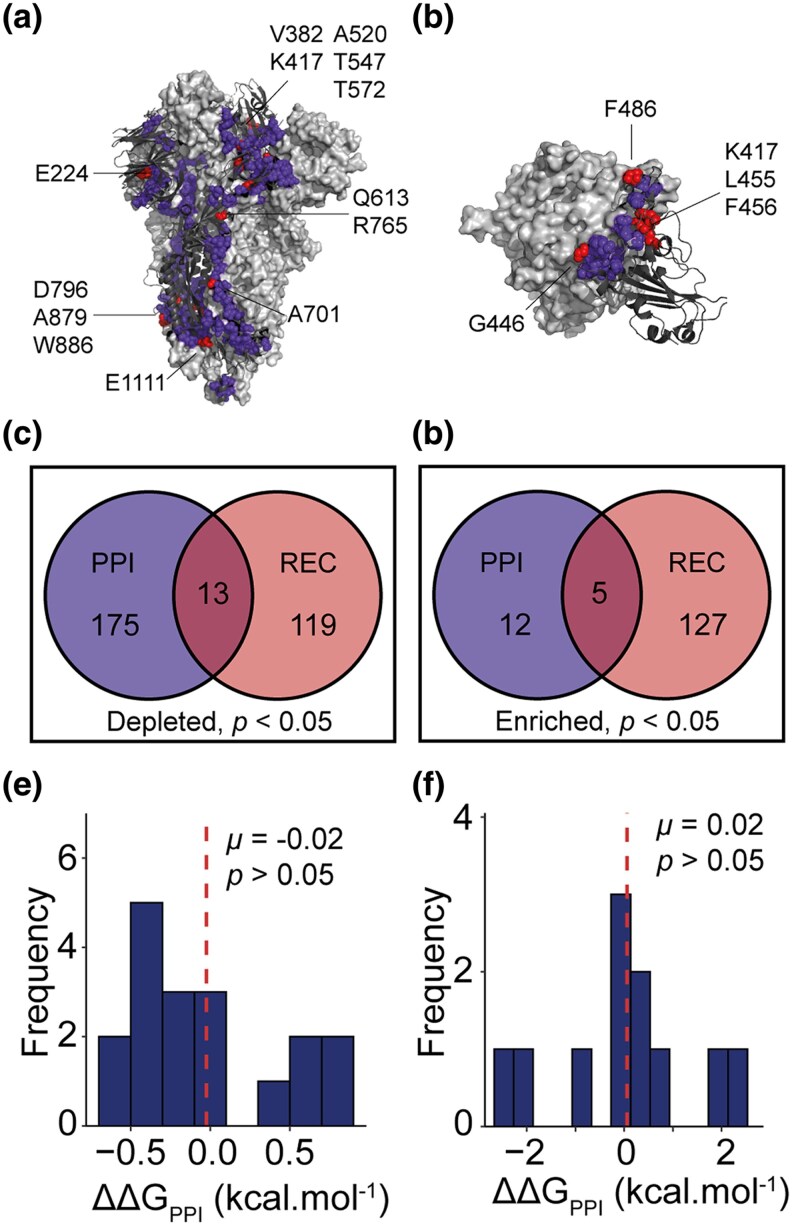
The effect of recurrent substitutions at protein–protein interfaces formed by the S protein. (a) The trimeric interface of the S protein complex (PDB: 6VXX). A single S protomer is shown as a dark grey cartoon. The residues interacting with the remaining two protomers (the light grey surface) are shown as spheres. Interacting residues that have undergone significant recurrent evolution are shown in red and the residue labelled. (b) Interface between the S protein RBD (dark grey cartoon) and the human angiotensin converting-enzyme 2 (hACE2; light grey surface) (PDB: 6M0J). Interface residues are shown as in (a). (c and d) Venn diagrams showing the results of hypergeometric tests between the protein–protein interface (PPI) residues with significant recurrence sites for the trimeric and hACE2 interfaces, respectively. (e and f) Histograms showing the effect of recurrent substitutions on the interaction energy of their respective interface (ΔΔ*G*_PPI_). The mean value (*μ*) is indicated with a dashed red line and *P*-values are the results of one sample *t*-tests.

To further investigate the functional implications of recurrent substitutions identified by RECUR, we next examined the protein–protein interfaces of the S protein, focusing on the prefusion trimeric interface and the human angiotensin-converting enzyme 2 (hACE2) receptor binding interface. To do this, residues at the trimeric and hACE2 interfaces were first identified using a distance threshold of less than 4 Å to an atom of the respective neighbouring protein (Fig. 11a, b, Files S7). An analysis of these sites revealed that there was a depletion of sites that have undergone significant recurrent evolution at the prefusion trimeric interface of the S protein complex (hypergeometric test, *P* < 0.05; Fig. 11c). In contrast, recurrently evolving sites were enriched at the interface with the hACE2 receptor (hypergeometric test, *P* < 0.05; Fig. 11d). Thus, recurrent evolution has been constrained at the trimeric interface, likely reflecting functional or structural limitations, whereas recurrent evolution is enriched at the immune-accessible hACE2 binding site of the S protein, where adaptive pressures such as host immune evasion drive repeated amino acid changes.

### Recurrent molecular evolution variably modulates hACE2 binding and immune evasion

Finally, we assessed the impact of the significant recurrent substitutions on both the trimeric prefusion and hACE2 interfaces. To do this, we calculated the change in the interaction energy (ΔΔ*G*_PPI_) upon *in silico* substitution (see Methods), where a negative ΔΔ*G*_PPI_ indicates interface stabilization and *vice versa*. Our analysis revealed that neither the recurrent substitutions at the trimeric interface nor those at the hACE2 interface significantly altered the stability of interaction (both one sample *t*-tests *P* > 0.05; and Fig. 11e, f). However, recurrent substitutions at the hACE2 interface exhibited significantly greater variability in their effects on interface stability compared to the trimeric interface (Levene's test, *P* < 0.05). Notably, four substitutions at the hACE2 interface had a |ΔΔ*G*_PPI_| > 1 kcal.mol^−1^. Two of these substitutions (F486P and K417N) destabilize the interface and have been associated with immune escape (Cao et al. 2022; McCallum et al. 2022; Qu et al. 2024) while the other two (P486F and N417K) are reversions back to the reference sequence. Together, these results show that while recurrent substitutions at the trimeric interface have limited impact on interface stability, those at the hACE2 interface are more variable, reflecting a balance between maintaining receptor binding and enabling immune evasion.

## Discussion

Recurrent evolution is a pervasive feature across the tree of life and a hallmark of adaptive evolution. At the molecular level, recurrent sequence changes provide powerful insights into the selective pressures shaping protein evolution. However, detecting such patterns easily and at scale remains challenging. Here, we present RECUR, an easy-to-use scalable tool that automatically detects recurrent amino acid substitutions from a protein (or a corresponding codon) multiple sequence alignment. RECUR enables the quantification of recurrent evolution across large datasets, revealing specific amino acid residues that are repeatedly observed across independent lineages and may be favoured by similar selective pressures. We showed that RECUR's recurrent detection is accurate and robust to realistic levels of tree inference error. We further applied RECUR to 123,126 surface glycoprotein sequences from SARS-CoV-2, identifying key sites that have undergone recurrent evolution during viral adaptation. This tool opens up new avenues for investigating the molecular basis of adaptation and constraint across diverse biological systems.

Our analyses demonstrate that RECUR's detection of recurrent evolution is robust to realistic levels of tree inference error. When comparing recurrent substitutions obtained from gene trees to those identified using a ‘true’ species tree, we observed a low false positive rate (3.3%), a small false negative rate (6.3%), and a high true positive rate (90.4%). Notably, tree inference from alignments can be affected by homoplasy, where the same descendent state arises independently in different lineages (Wake 1991; Crispell et al. 2019). If a tree reconstruction method interprets these independently derived states as shared ancestry, it can cluster those sequences together incorrectly (Philippe et al. 2011). In such scenarios, sequence similarity due to convergence can lead to underestimation of recurrent evolution because sequences that share the same derived state but are distantly related are not recognized as independent events by the inferred tree. This effect explains why false positives remain low in our gene tree analysis and highlights the importance of considering tree inference limitations. The low false positive rate is particularly important for downstream applications, as it ensures that sites identified as recurrent are reliable targets for experimental interrogation.

Since tree inference error is common in phylogenetic analyses, RECUR was designed to allow users to input a precomputed constraint tree that restricts the topological search during phylogenetic inference. This functionality enables users to leverage additional information to infer the constraint tree that may not be available in the alignment under consideration. For example, users may wish to constrain the topology of the tree to match a known species tree when analysing orthologous sequences derived from those species. While the use of a constraint tree does not eliminate tree inference error, it can help reduce its impact when reliable prior information is available.

To contextualize RECUR's performance, we compared it to an existing phylogenetic tool, TreeTime, which can infer branch-specific substitutions across a phylogeny. Using simulated datasets, we observed a high level of concordance between the two approaches, with 99.7% agreement in the recurrent substitutions identified. The small number of differences were attributable to the underlying ancestral sequence reconstruction methods, as RECUR relies on IQ-TREE and TimeTree implements its own reconstruction algorithm. Importantly, RECUR addresses a key methodological gap by directly and automatically identifying recurrent substitutions, a functionality not provided by TreeTime, and by integrating a novel statistical assessment via Monte Carlo simulation. This removes the need for users to manually extract substitutions or have expertise in handling phylogenetic trees, ancestral reconstructions, and sequence simulators. As a result, RECUR provides a streamlined and reproducible workflow for detecting statistically significant recurrent substitutions, while maintaining strong agreement with existing methods for substitution inference.

To demonstrate the performance characteristics of RECUR we examined how the runtime and memory use of the method is influenced by the number of sequences, the sequence length, and the extent of recurrence present in the sequences under consideration. Larger numbers of sequences, longer alignments, and higher rates of recurrence each result in increased computational demand. The SARS-CoV-2 surface glycoprotein used to evaluate these performance characteristics has a high mutation rate and is substantially longer than the average eukaryotic or bacterial protein (S protein length = ∼1,300, average prokaryotic protein length = ∼300, average eukaryotic protein length = ∼500 (Tiessen et al. 2012)), thus the results presented here represent a conservative estimate of what can be expected by a user when running RECUR. Furthermore, our benchmarking revealed that most of RECUR's runtime is consumed by the phylogenetic tree inference step, not by the recurrence detection algorithm itself. Thus, improvements in the scalability of these methods will also result in further improvements in the performance characteristics of RECUR.

To provide an example of a real-world use of RECUR's, we applied the method to the complete set of nonredundant SARS-CoV-2 surface glycoprotein sequences available in NCBI. This analysis revealed widespread recurrent evolution across the length of the protein. Furthermore, we uncovered a significant enrichment of sites that have undergone recurrent evolution in the S1 subunit compared to a significant depletion in the S2 subunit. This aligns with the functional and structural differences between these two regions of the S protein. The S1 subunit, contains the receptor binding domain and is essential for viral attachment to the hACE2 receptor, making it a hotspot for adaptive evolution as the virus evolves to concurrently optimize binding affinity and evade the immune system (Tian et al. 2021). Given the trade-offs between these factors, there are high levels of recurrent reversion substitutions in this region of the S protein as the virus undergoes adaption during cycles of selection pressure. Additionally, the S1 subunit contains the N-terminal domain, which contributes to antigenicity and hence adaptative evolution has repeatedly evolved mechanisms for immune escape in this region (McCallum et al. 2021b). Finally, although depleted, sites under recurrent evolution were also identified in the S2 subunit. These substitutions may benefit the virus through aiding immune escape through allosteric effects on S1 epitopes (Kumar et al. 2023). Importantly, this analysis has identified many sequence changes for which we could find no existing experimental interrogation in the literature, but which have repeatedly evolved during the radiation of SARS-CoV-2. Future functional interrogation of these sites may reveal novel mechanisms of viral adaptation and uncover previously undiscovered determinants of viral fitness. While our analysis is not intended to be exhaustive, it highlights RECUR's utility as a tool for understanding the evolution of a protein and for the identification of residues of interest for downstream functional studies.

The RECUR method makes extensive use of the IQ-TREE 2 software package (Minh et al. 2020). IQ-TREE 2 was chosen over other phylogenetic inference software for several reasons. First, IQ-TREE 2 is a mature, well-developed, actively maintained and thoroughly benchmarked software package that has the capability to carry out many steps required in the RECUR algorithms. These steps include model selection, tree inference, ancestral state reconstruction, and alignment simulation. It is not possible to achieve all of these steps using comparable software such as RAxML (Stamatakis 2014) or PhyML (Guindon and Gascuel 2003). Thus, use of IQ-TREE 2 reduces the number of dependencies required by RECUR. Second, IQ-TREE performs very well against all competitor methods in terms of both speed and accuracy (Zhou et al. 2018). Third, IQ-TREE 2 can analyse alignments with thousands of sequences allowing RECUR to also scale to this number of sequences. Together, these features ensure that RECUR takes advantage of the latest advances in phylogenetic inference and is both robust and scalable.

Analyses of positive selection and recurrent evolution capture distinct signals of molecular adaptation. Positive selection tests quantify the rate of sequence change, specifically the ratio of the rate of nonsynonymous to synonymous substitution (dN/dS), identifying sites where the rate of nonsynonymous substitution is higher than expected. In contrast, RECUR focuses on the recurrence of substitutions, identifying specific substitutions that have higher levels of recurrence than expected given the number of sequences sampled, the phylogenetic relationship of those sequences, and the best fitting model of sequence evolution identified from the input alignment. Despite these differences, we observed a significant overlap in the sites detected by both approaches, suggesting that many sites exhibit multiple signals of adaptive evolution. This observation is consistent with a previous study of adaptative evolution in the photosystem genes of flowering plants, which also showed that sites evolving under strong purifying selection were depleted in recurrent evolution (Robbins and Kelly 2024). Differences in the sites that are identified by each method reflect their unique detection criteria. For example, RECUR accounts for the expected likelihood of different amino acid substitutions under the inferred model of evolution, such that highly probable substitutions must recur many times to be considered significant, whereas rarer, less likely substitutions can be identified with fewer independent observations—an aspect not considered by conventional positive selection tests. Given these findings, RECUR can serve as a valuable tool for studying adaptive evolution, either as a standalone approach or in conjunction with positive selection analyses, providing a more comprehensive view of evolutionary dynamics across proteins.

In summary, RECUR is a comprehensive tool for identifying recurrent amino acid substitutions from multiple sequence alignments. We anticipate that RECUR will serve as a resource for understanding the molecular basis of convergent phenotypes as well as generating hypotheses and guiding experimental testing of protein function.

## Materials and methods

### Implementation

RECUR is a Python application that leverages IQ-TREE 2 (Minh et al. 2020) as an external dependency and requires Python 3.9 or higher. The application utilizes DendroPy (Sukumaran and Holder 2010) to extract ancestor-descendent sequence pairs from unrooted trees in Newick format. RECUR achieves high performance through the use of NumPy (Harris et al. 2020) and multiprocessing, particularly in key steps such as the substitution analysis of Monte Carlo simulated protein sequences. In this process, the ancestor-descendent sequence pairs are first converted into numerical representations and then into NumPy arrays for element-wise comparison at each residue site. Multiprocessing is employed to further speed up these operations. RECUR includes a Linux version of the IQ-TREE 2 binary, enabling it to run as a standalone application on Linux systems. For Windows and macOS users, IQ-TREE 2 must be preinstalled. Regardless of the operating system, it is recommended to run RECUR within a conda environment or a Docker container for compatibility. Full instructions on the installation and implementation of RECUR can be found at https://github.com/OrthoFinder/RECUR.

### Tree inference and model selection

RECUR takes a codon or protein multiple sequence alignment as input. The alignment is evaluated to identify the best-fitting model of sequence evolution using ModelFinder implemented in IQ-TREE 2 (Kalyaanamoorthy et al. 2017). Using this model, a maximum likelihood phylogenetic tree is constructed with IQ-TREE 2, with optional calculation of branch support values using IQ-TREE's ultrafast bootstrapping method with 1,000 replicates (Hoang et al. 2018). Importantly, bootstrap support values are not required for RECUR's recurrent substitution analysis and are provided solely for optional user assessment of the inferred phylogeny. Enabling bootstrap inference increases computational and runtime demands. Alternatively, if the user has already precomputed a phylogenetic tree or preselected a model of sequence evolution, then these can be provided as inputs to RECUR and be used in all analysis steps. The tree is then rooted on a user-defined outgroup which can comprise a single sequence or a list of outgroup sequences. In the case where the supplied outgroup sequences are not monophyletic in the tree, the smallest possible clade comprising the full set of outgroup sequences will be selected as the outgroup.

### Identifying recurrent amino acid substitutions

Ancestral sequence reconstructions are inferred for every node in the phylogeny using the best-fitting model of sequence evolution and the inferred maximum likelihood tree using the ancestral state reconstruction method implemented in IQ-TREE 2 (Minh et al. 2020). To correctly infer gaps in ancestral sequences (which IQ-TREE 2 currently cannot implement) the multiple sequence alignment is converted into binary sequences, with 0 and 1 representing gapped and nongapped sites, respectively. As with the biological sequences, the ancestral reconstructions of the binary sequences are inferred for every node in the phylogeny using the inferred maximum likelihood tree (for which the branch lengths are re-inferred) and the general time reversible model for binary data model (GTR2) which allows for unequal state frequencies. The user may optionally extend the GTR2 model by including a proportion of invariant sites (+I), rate heterogeneity among site (+G), or both (+I + G). The positions of the inferred 0 values, i.e. gaps, in the ancestral sequences are then mapped onto the biological sequences to complete ancestral sequence reconstruction with indel estimation.

If a codon alignment was provided, both the inferred and extant sequences are then translated into protein sequences using the standard genetic code as default. The user can also specify alternative NCBI genetic codes to translate DNA sequences if required. For each residue in the protein sequence, amino acid substitutions are tabulated by analysing the residue identity for all ancestor-descendent sequences at every branch in the subtree that excludes outgroup sequences. A list of recurrent substitutions, i.e. substitutions that occurred more than once at a given site during the evolution of the sequence set, is then compiled.

### Identification of significant recurrent sites

To assess whether recurrent amino acid substitutions occur more frequently than expected by chance, RECUR performs a Monte Carlo simulation of sequence evolution. Specifically, the inferred phylogeny, model of evolution, and root sequence of the subtree of interest are then used to simulate codon/protein sequence evolution *M* times using the alisim function in IQ-TREE 2 (Ly-Trong et al. 2023), where *M* is automatically calculated based on the number of tests to ensure sufficient resolution for detecting significance after multiple testing correction. As explained above, the gaps are mapped onto each simulated alignment. This prevents the overestimation of substitution recurrence. The amino acid substitutions that have occurred in each of the simulated sequence alignments are then identified using the method outlined above. For each of the recurrent substitutions identified for the real alignment, RECUR assesses the number of times that site specific substitution has occurred at the same or greater frequency in the simulated alignments. A *P*-value describing the probability that the recurrence of that site specific amino acid substitution having occurred by chance (*p*_i_) is then assigned using the following equation:


$$p_{i}=\frac{\sum_{\;j=1}^{M}\;C_{ij}+1}{M+1}\;,\;i=1,2,\;\ldots ,N$$

$$C_{ij}=\;\{\begin{array}{c} 1\;\;\;\;\;\;R_{i(x\to y)j}^{mcs}\geq \;R_{i(x\to y)}\\ 0\;\;\;\;\;\;R_{i(x\to y)j}^{mcs}<\;R_{i(x\to y)} \end{array}\;\;\mathrm{where}\;R_{i}>1$$

where *i* is the *i*th residue, *N* is the number of residues in the protein alignment, *j* is *j*th sequence simulation, *M* is the number of sequence simulations run, *C_ij_* is the piecewise function, *R*_*i(x*→*y)*_ is the recurrence of amino acid substitution *x* → *y* at the *i*th site in the real phylogeny, and *R^mcs^*_*i(x*→*y)j*_ is the recurrence of amino acid substitution *x* → *y* at the *i*th site in the *j*th simulation. Finally, *P*-values are corrected for multiple testing using a method that is automatically selected based on the number of tests: Bonferroni for fewer than 50 tests, Holm or FDR for intermediate sizes, and adaptive FDR methods (e.g. fdr_tsbh) for larger test sets unless a correction method is specified by the user.

### Assessing the accuracy of RECUR's recurrence substitution detection

To validate the accuracy of RECUR's recurrent substitution detection algorithm, we simulated the evolution of 1,000 human protein-coding genes. To do this, the current human coding DNA sequences were downloaded from NCBI on the 29 July 2025, and 1,000 genes were randomly sampled. For each gene, a corresponding phylogenetic tree was simulated using the alisim function in IQ-TREE 2 (Ly-Trong et al. 2022). Minimum and maximum branch lengths were fixed at 0.001 and 0.1, respectively, while the mean branch length was drawn from a normal distribution with a mean of 0.01 ± 0.02. The number of taxa per tree was sampled from a normal distribution of 40 ± 10.

Each sampled gene was then evolved along its corresponding tree, after rooting on a random taxon, using phastSim (De Maio et al. 2022). We specifically used phastSim because it records the exact substitutions introduced during the simulation, providing a known ground truth for evaluating RECUR's detection accuracy. To restrict evaluation to RECUR's recurrence detection algorithm, both external and internal sequences generated by phastSim, together with the corresponding tree and outgroup taxa, were provided as input to RECUR. Internal sequences were reconstructed from phastSim's annotated Newick files, ensuring that RECUR operated on the exact simulated ancestral states.

### Benchmarking RECUR against TreeTime for recurrence substitution detection

To further evaluate RECUR's ability to identify recurrent substitutions, we benchmarked its performance against TreeTime, an existing tool that can infer substitutions occurring along a phylogeny. To do this, we sampled 1,000 human proteins and generated their corresponding phylogenies and alignments using IQ-TREE's *alisim* function with the same parameters described above (Ly-Trong et al. 2022). RECUR was applied to each alignment using the associated phylogeny, with a random taxon specified as the outgroup. TreeTime was then run on the same alignments and phylogenies and recurrent substitutions were identified from the output list of substitutions inferred to have occurred along branches of the tree. To ensure comparability with RECUR, substitutions occurring along the outgroup branch were excluded from TreeTime's results as RECUR does not analyse substitutions on the outgroup lineage.

### Assessing the effect of tree inference error on recurrence detection

To evaluate the impact of tree inference error on the detection of recurrent substitutions, we analysed a dataset of 69 highly conserved plastid-encoded genes from 773 angiosperm species. Multiple sequence alignments and the corresponding species tree, which was generated from the concatenation of all 69 genes, were obtained from (Robbins and Kelly 2023). RECUR was run on all protein multiple sequence alignments under two conditions: using a species tree as a fixed constraint and without specifying a tree (e.g. allowing RECUR to infer a tree from the alignment). For each gene, we calculated the percentage difference in recurrent substitutions between the two conditions, which quantifies the relative change in recurrence calls attributable to tree inference (File S8). To measure the extent of tree inference error, we computed the normalized Robinson-Foulds distance between gene trees and the species tree using the ETE3 software package (Huerta-Cepas et al. 2016).

### SARS-CoV-2 surface glycoprotein dataset retrieval and curation

A dataset comprising all available SARS-CoV-2 surface glycoprotein coding sequences derived from human hosts was downloaded from NCBI on 29th July 2024 (*n* = 3,934,602). The dataset was filtered to remove sequences shorter than 3,800 bp, sequences with ambiguous characters and sequences without start or stop codons. The coding sequences were then translated into protein using the standard genetic code and aligned using FAMSA (Deorowicz et al. 2016). The alignment was trimmed to remove columns with >50% gaps and redundant sequences were removed resulting in an alignment containing 123,126 SARS-CoV-2 surface glycoprotein sequences with 1,273 columns. Finally, 15 sites known to cause problems with phylogenetic analyses were obtained from https://genome.ucsc.edu/cgi-bin/hgTables and masked in the alignment. The final alignment is provided in File S9. The sequence identical to the Wuhan-1 SARS-CoV-2 surface glycoprotein (accession: MT582461.1) was used as the outgroup.

### Computing runtime and memory usage

To demonstrate the runtime characteristics, the 123,126 sequence multiple sequence alignment was randomly sampled, while preserving the outgroup species, to create 10 alignments with 8, 16, 32, 64, 128, 256, 512 and 1,024 sequences, respectively, totalling 80 alignments. RECUR was run on each alignment with six input option combinations: no constraint tree, no branch support testing and no evolutionary model; no constraint tree, no branch support testing but with an evolutionary model; no constraint tree, no evolutionary model but with branch support testing; no constraint tree but both branch support testing and an evolutionary model; a constraint tree and no evolutionary model (branch support testing not applicable); and finally a constraint tree and an evolutionary model (branch support testing not applicable). The evolutionary model and constraint trees used were those obtained from the RECUR output under the first input scenario. Each run was timed, and the maximum proportional set size (PSS) was recorded. These tests were conducted on an AMD EPYC 7742 64-Core Processor (2.25 GHz, x86_64 architecture) and given eight threads.

### Analysing the complete 123,126 surface glycoprotein sequence dataset

Although RECUR itself scales efficiently to datasets containing thousands of sequences, the principal computational challenge at this scale arises from phylogenetic tree inference. For the complete dataset of 123,126 SARS-CoV-2 surface glycoprotein sequences, conventional maximum likelihood tree inference with IQ-TREE 2 was not feasible. Instead, a maximum likelihood constraint tree was inferred using Very Fast Tree (Piñeiro et al. 2020), which is specifically designed for large datasets. Additionally, the best fitting model of sequence evolution and the averaged model parameters, HIVw + F + I[0.24] + G4[0.61], was taken from the runtime and memory usage runs for the 1,024 sequence alignment runs above. By constraining the tree and the model of sequence evolution RECUR is thus able to analyse the complete dataset, and Bonferroni correction was applied to adjust *P*-values for multiple testing.

### Calculating the weighted average likelihood of substitution per site

The likelihood of a substitution was defined by the rate matrix of the best fitting model of sequence evolution identified, HIVw (Nickle et al. 2007). For exclusively positive selection sites, the average likelihood of substitution weighted by the substitutions’ recurrence was calculated by summing the product of recurrence and HIVw across all substitutions at a site and dividing by the total number of substitutions. Meanwhile, for exclusively recurrently evolving sites the calculation only considered significant substitutions identified by RECUR.

### Residue solvent accessibility analysis

The relative solvent accessibility (RSA%) was determined for each residue in the closed S protein trimeric structure (PDB: 6VXX) using the GetArea web server (https://curie.utmb.edu/getarea.html, accessed on 24th February 2025) (File S10).

### Protein–protein interface analysis

Protein–protein interface residues were identified using the EMPL-EBI PDBsum database. To analyse the trimeric S protein interface, the closed (PDB: 6VXX) and open (PDB: 7W92) structures were utilized, while the hACE2 interface was examined using the 6M0J structure (File S7). The effects of recurrent substitutions were assessed by introducing single amino acid substitutions using PyMol. Then, the change in Gibb's free energy of interaction (Δ*G*_PPI_) was calculated using BAlaS at the respective interface for both the wild-type and mutated structure (Wood et al. 2020). From these, the change in Δ*G*_PPI_ can be calculated, where a negative value indicates a strengthening of the interface, and a positive value indicates a weakening of the interface (File S6).

## Supplementary Material

msag036_Supplementary_Data

## Data Availability

The source code is available on the RECUR GitHub repository (https://github.com/OrthoFinder/RECUR). All data used in this study is provided in the material.
