# Supplementary material for: RECUR: identifying recurrent amino acid substitutions from multiple sequence alignments
Source: Mol Biol Evol. 2026 Feb 10;43(2):msag036. doi: 10.1093/molbev/msag036 (PMC12930092; doi:10.1093/molbev/msag036)
Supplement: msag036_Supplementary_Data [file msag036_supplementary_data.zip › Supplementary_Figures.docx]

***Supplementary Figure S1***

***
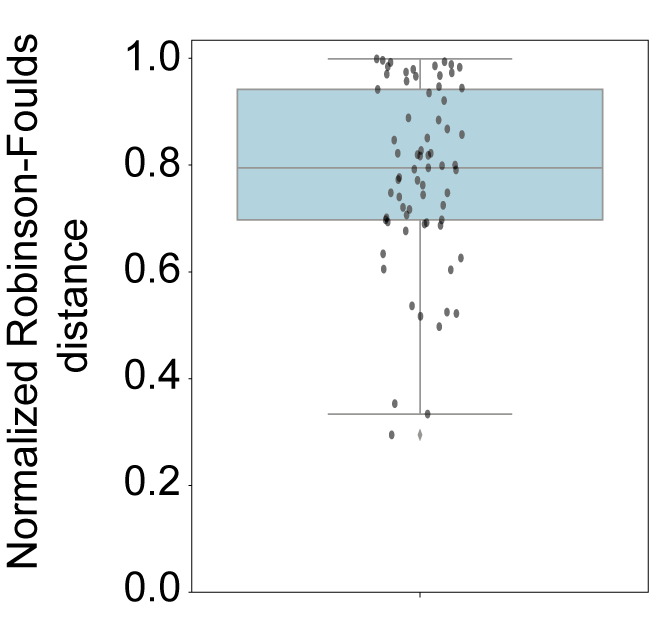
***

**Supplementary Figure S1.** Boxplot showing the topological distances between individual gene trees and the species tree. Normalized Robinson-Foulds (nRF) distances were calculated between each gene tree (n = 69) and the species tree inferred from the concatenated alignment.

***Supplementary Figure S2***

***
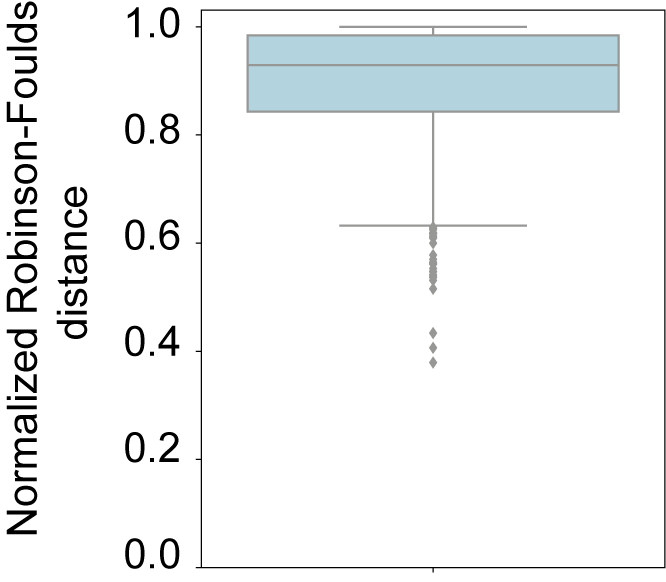
***

**Supplementary Figure S2.** Boxplot showing the topological distances among the 69 individual gene trees. Normalized Robinson–Foulds (nRF) distances were calculated for all pairwise comparisons between the 69 gene trees (n = 2,346).
